# Genetic and phenotypic associations between root architecture, arbuscular mycorrhizal fungi colonisation and low phosphate tolerance in strawberry (*Fragaria* × *ananassa*)

**DOI:** 10.1186/s12870-020-02347-x

**Published:** 2020-04-09

**Authors:** Helen Maria Cockerton, Bo Li, Eleftheria Stavridou, Abigail Johnson, Amanda Karlström, Andrew Douglas Armitage, Ana Martinez-Crucis, Lorena Galiano-Arjona, Nicola Harrison, Nuria Barber-Pérez, Magdalena Cobo-Medina, Richard Jonathan Harrison

**Affiliations:** 1 NIAB EMR, New Road, East Malling, Kent, ME19 6BJ UK; 2 University of the West of England, Bristol, UK; 3 NRI, University of Greenwich, Kent, UK; 4 AHDB, Agriculture and Horticulture Development Board, Stoneleigh Park, Kenilworth, Warwickshire CV8 2TL UK

**Keywords:** QTL, Macronutrients, Symbiont, Network, Trade-off, Solidity, Rhizotron, Length distribution, AMF, Image analysis

## Abstract

**Background:**

Phosphate is an essential plant macronutrient required to achieve maximum crop yield. Roots are able to uptake soil phosphate from the immediate root area, thus creating a nutrient depletion zone. Many plants are able to exploit phosphate from beyond this root nutrient depletion zone through symbiotic association with Arbuscular Mycorrhizal Fungi (AMF). Here we characterise the relationship between root architecture, AMF association and low phosphate tolerance in strawberries. The contrasting root architecture in the parental strawberry cultivars ‘Redgauntlet’ and ‘Hapil’ was studied through a mapping population of 168 progeny. Low phosphate tolerance and AMF association was quantified for each genotype to allow assessment of the phenotypic and genotypic relationships between traits.

**Results:**

A “phosphate scavenging” root phenotype where individuals exhibit a high proportion of surface lateral roots was associated with a reduction in root system size across genotypes. A genetic correlation between “root system size” traits was observed with a network of pleiotropic QTL found to represent five “root system size” traits. By contrast, average root diameter and the distribution of roots appeared to be under two discrete methods of genetic control. A total of 18 QTL were associated with plant traits, 4 of which were associated with solidity that explained 46% of the observed variation. Investigations into the relationship between AMF association and root architecture found that a higher root density was associated with greater AMF colonisation across genotypes. However, no phenotypic correlation or genotypic association was found between low phosphate tolerance and the propensity for AMF association, nor root architectural traits when plants are grown under optimal nutrient conditions.

**Conclusions:**

Understanding the genetic relationships underpinning phosphate capture can inform the breeding of strawberry varieties with better nutrient use efficiency. Solid root systems were associated with greater AMF colonisation. However, low P-tolerance was not phenotypically or genotypically associated with root architecture traits in strawberry plants. Furthermore, a trade-off was observed between root system size and root architecture type, highlighting the energetic costs associated with a “phosphate scavenging” root architecture.

## Background

Phosphate is the most limiting plant macronutrient in agriculture with an estimated 30–40% of arable land restricted by poor phosphate bioavailability [1]. Enhancing the capacity for crops to utilise both pre-existing and supplementary phosphate is one strategy that can be used to lower fertiliser requirements. Greater phosphate use efficiency (PUE) may be achieved through breeding crops with optimised root architecture and an increased propensity to form mycorrhizal associations [2]. Both such traits enhance external PUE through improving the uptake of phosphate and thus promoting enhanced plant yield, as opposed to improving internal PUE through improved conservation and remobilisation of existing phosphorus stores [3].

Root system architecture is known to play an important role in phosphate acquisition, as finer roots have an increased capacity to absorb nutrients [4]. Greater lateral root branching and higher root hair density are associated with greater phosphate uptake [5–7]. Roots secrete organic acids which mobilize phosphate in the immediate root area thus allowing phosphate uptake and creating a nutrient depletion zone [8]. Symbiotic association with Arbuscular Mycorrhizal Fungi (AMF) however, can allow a plant to access phosphate from beyond the root nutrient depletion zone through the exploitation of the expansive AMF extraradical hyphal network [9]. Plants are able to use mycorrhizae to functionally substitute the role of plant root hairs in phosphate uptake [10]. Such symbiotic associations are highly advantageous under nutrient limiting conditions; indeed, the majority of higher terrestrial plants form mycorrhizal associations [11].

The study of phosphate uptake in the strawberry cultivar ‘Hapil’ showed a greater demand for phosphate by flowers in the reproductive phase of development and this demand was not met by the reallocation of existing phosphate, this additional phosphate was in fact acquired from the environment at a rate which exceeded the capacity of the root system [12] leading to a suggestion of the necessity for AMF association in strawberry production. AMF colonisation has been found to increase the yield of class one strawberry fruit production in coir [13]. Further to this, phosphate solubilising bacteria (PSB) have been shown to enhance the level of bioavailable phosphate for plant uptake in soil pot experiments and led to an increase in strawberry yields [14]. Indeed, AMF have been found to act synergistically with PSB in the presence of high phosphate and competitively under low phosphate conditions [15], indicating the importance of the microbial community for optimum phosphate acquisition.

AMF symbiotic association has been shown to affect multiple strawberry traits alongside enhanced phosphate uptake under deficit conditions [16]. AMF have been found to restore and even enhance strawberry plant biomass under drought stress [17], increase vegetative reproduction [18, 19], increase fruit yield and number [20] and enhance anthocyanins and phenolic production in strawberry fruit [21, 22]. Furthermore, AMF colonisation was found to induce strawberry root architecture changes (through an increase in root branching) which was associated with greater resistance to *Phytophthora fragariae* [23]. Cultivar-specific AMF interactions have been reported in strawberry, such interactions indicate that the genetic components controlling host-mycorrhizal association may be harnessed through breeding strategies [23–25].

Root architecture is starting to receive greater attention as a mechanism to improve both crop yield and quality [26]. Indeed, the production of high nutrient efficient root systems have been suggested to be the key to a second green revolution [7]. Roots may exhibit a modular plastic response to soil heterogeneity with the ability to proliferate and enhance ion uptake only in nutrient rich zones [27]. By contrast, the whole root system may alter in response to average nutrient levels leading to among-plant variability. Homogeneously low phosphate, has been found to reduce primary root growth, increase lateral density and root length [28]. Furthermore, different genotype-specific root strategies have been observed in common bean where trade-offs exist between deep root phenotypes associated with drought tolerance and lateral shallow roots associated with low phosphate tolerance [29].

Increasing ion uptake through physiological root plasticity is an effective strategy for uptake of mobile ions such as nitrate. However, morphological plasticity is more costly and must be used to enhance the uptake of immobile ions such as phosphate [27]. The cost of implementing morphological plasticity is high, therefore it can only be considered as a desirable nutrient acquisition strategy where the temporal and spatial components of a nutrient patch are rich and predictable. Plants have been reported to increase lateral root growth in response to low phosphate environments [28, 30] and such changes to root architecture have been found to increase phosphate uptake from both heterogeneous localised patches and homogeneous phosphorus-rich surface soil [31]. It is clear that root morphology is directly linked to a plant’s phosphate capture potential, particularly for non-mycorrhizal plants; for example, *Arabidopsis thaliana arx4* mutants with lower lateral root production showed a reduced competitive ability to capture phosphate ions [32].

In this study, we aim to quantify the genotypic components controlling root architecture, AMF association and low phosphate tolerance in strawberry and look to establish relationships between traits. Fundamentally, this work will enhance breeding resources towards the improvement of phosphate acquisition in octoploid strawberry.

## Results

### Description of parental and progeny root architecture

**Fig. 1:**
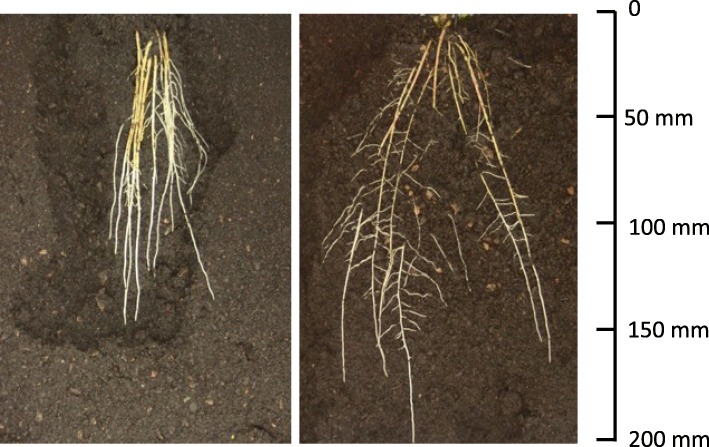
Strawberry root systems grown in rhizotrons a) ‘Hapil’ cultivar displaying roots with a low convex area and high average root diameter b) ‘Redgauntlet’ cultivar high convex area and low average root diameter

**Fig. 2:**
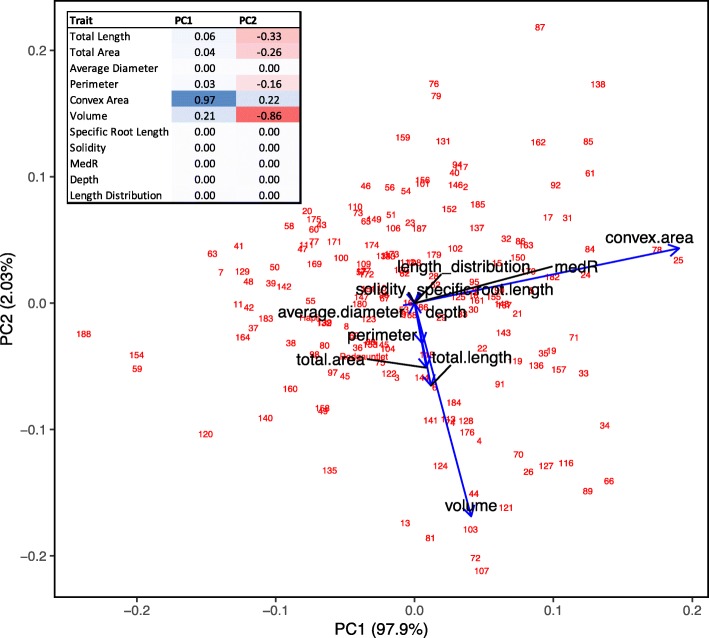
Principal Component Analysis (PCA) biplot of average genotype scores for the ‘Redgauntlet’ x ‘Hapil’ mapping population root architecture. Red numbers represent genotype numbers, Blue arrow represent the contribution and direction of root trait contribution on PC1 and PC2. Percentage figures along axis indicate the proportion of variation explained by PC. Figures in the table represent correlation coefficients between PC’s and root traits, blue shading represents a positive correlation, red a negative correlation, strength of colour indicates strength of relationship

**Fig. 3:**
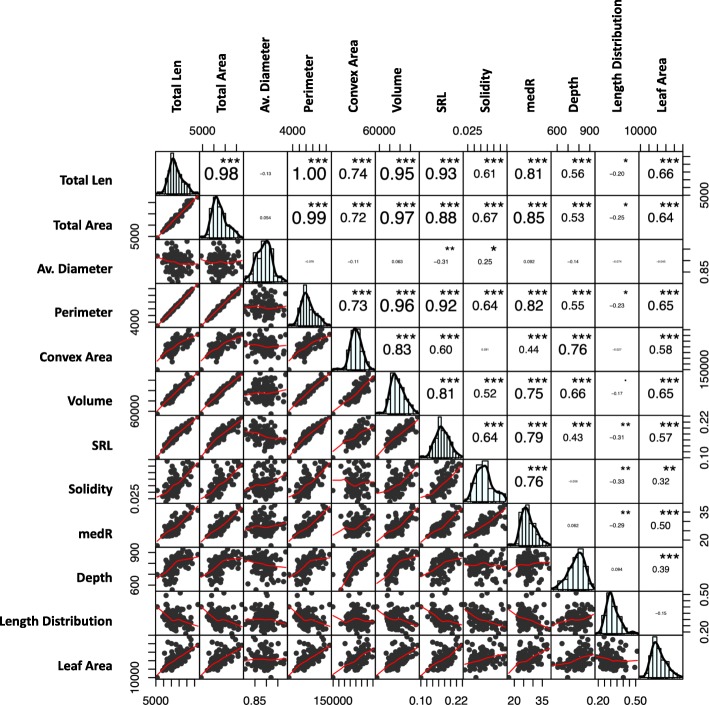
Phenotypic Correlation matrix between plant architectural traits. Numbers represent Pearson’s correlation coefficients size of font indicates the magnitude of the value. *p values* are denoted by stars: *** < 0.001, ** < 0.01, * < 0.05, ^.^ < 0.1

The two parental strawberry cultivars ‘Redgauntlet’ and ‘Hapil’ demonstrated contrasting root architecture when propagated as misted tips; ‘Redgauntlet’ roots had a small average root diameter whilst ‘Hapil’ roots had a relatively large average root diameter (Supp. Table 1). Furthermore, where runner plants were pinned down into rhizotrons and remained attached to the maternal plant, ‘Redgauntlet’ and ‘Hapil’ roots showed a greater contrast in root architecture. ‘Redgauntlet’ had a sparse and deep root system whereas ‘Hapil’ had a dense and shallow root system. In fact, all studied traits with the exception of median number of roots (medR) and length distribution were significantly different between parental root systems (Fig. 1, Table 1). By contrast, the variation in root architecture within the mapping population could be predominantly described by variation in the space the root network explores (convex area; Fig. 2: PC1 & convex area *r* = 0.97). Traits representing metrics of plant size were highly positively correlated (volume, root area, leaf area, length and perimeter; Fig. 3). Weak negative, but significant, correlations were observed between multiple plant size metrics and length distribution (*r* − 0.20 to − 0.33), indicating a trade-off between overall plant size and allocation of a greater proportion of resources to top surface roots.

**Table 1:** Parental root architecture trait means and significance values associated with ANOVA test. Units are pixel number unless otherwise stated. SRL – Specific root length, medR- medium root number. Coefficient of variation values (CV) are provided for each trait. *p values* are denoted by stars: *** < 0.001, ** < 0.01, * < 0.05

	Hapil	Hapil (CV)	Redgauntlet	Redgauntlet (CV)	Difference among parents
Total length	8036	59.2	14,214	52.4	***
Total area	10,303	54.6	15,487	48.4	**
Average diameter	1.596	21.5	1.293	11.3	***
Perimeter	3918	58.2	7014	51.5	***
Convex area	72,238	70.0	176,890	42.0	***
Volume	84,951	45.9	110,934	36.5	*
SRL (Length: Area)	0.091	38.8	0.121	28.1	**
Solidity (Convex area: Area)	0.1904	54.6	0.0892	28.2	***
medR (Frequency)	32	27.1	33	31.1	ns
Depth	425.4	33.1	633.9	18.0	***
Length distribution (Root area top 1/3: Root area bottom 2/3)	0.773	34.0	0.856	23.5	ns

### Plant architecture QTL

**Fig. 4:**
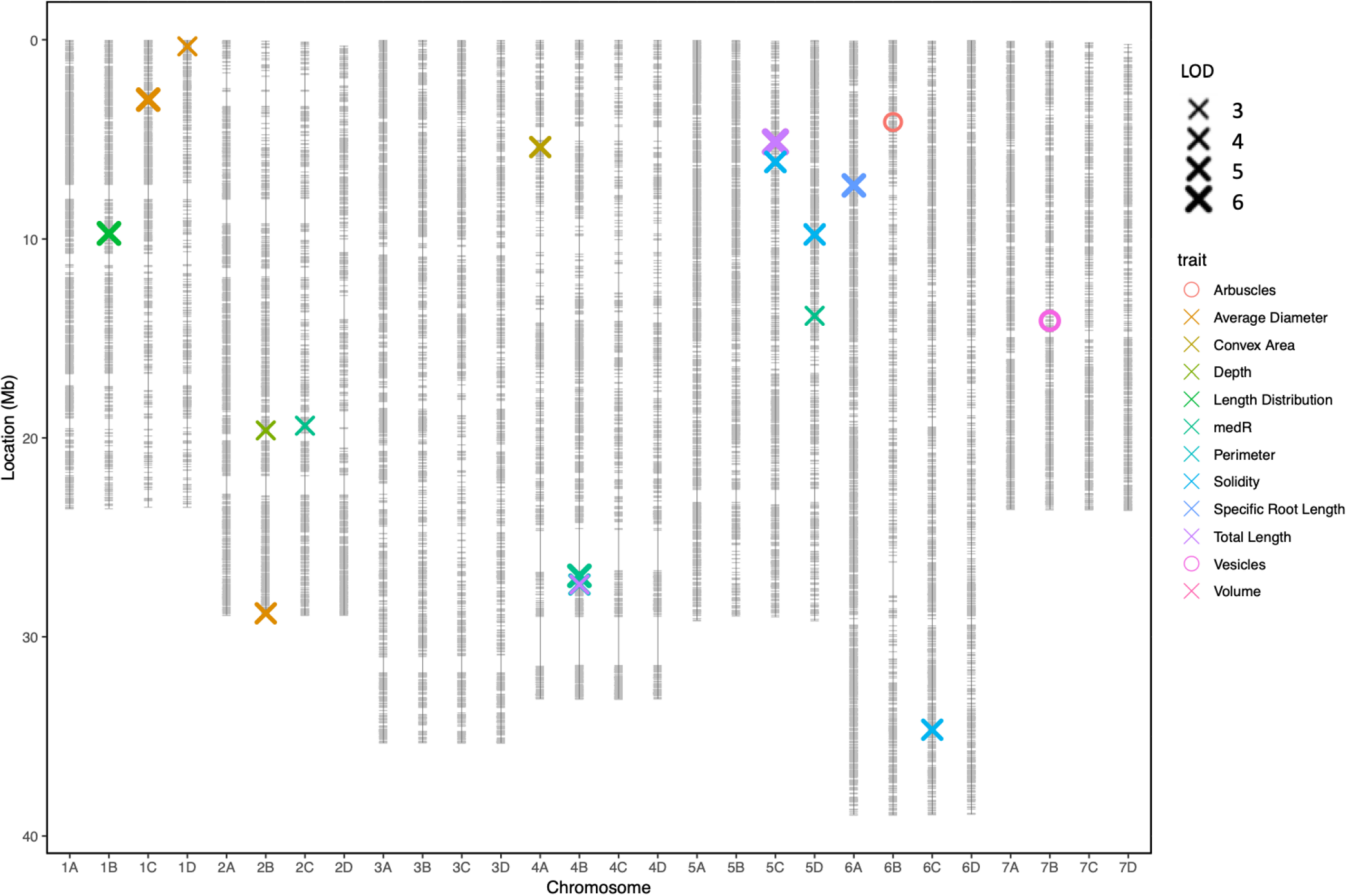
Linkage map displaying 35,154 marker positions (grey) in Mb for the 28 linkage groups of octoploid strawberry (1A-7D). Marker positions are scaled to *F. vesca* genome. Point shape denotes quantitative trait loci associated with each type of trait studied across the ‘Redgauntlet’ x ‘Hapil’ octoploid strawberry mapping population. Colours denote trait measures as detailed in the legend. Points are weighted based on significance with thicker lines representing greater significance. Line width denotes LOD values

**Fig. 5:**
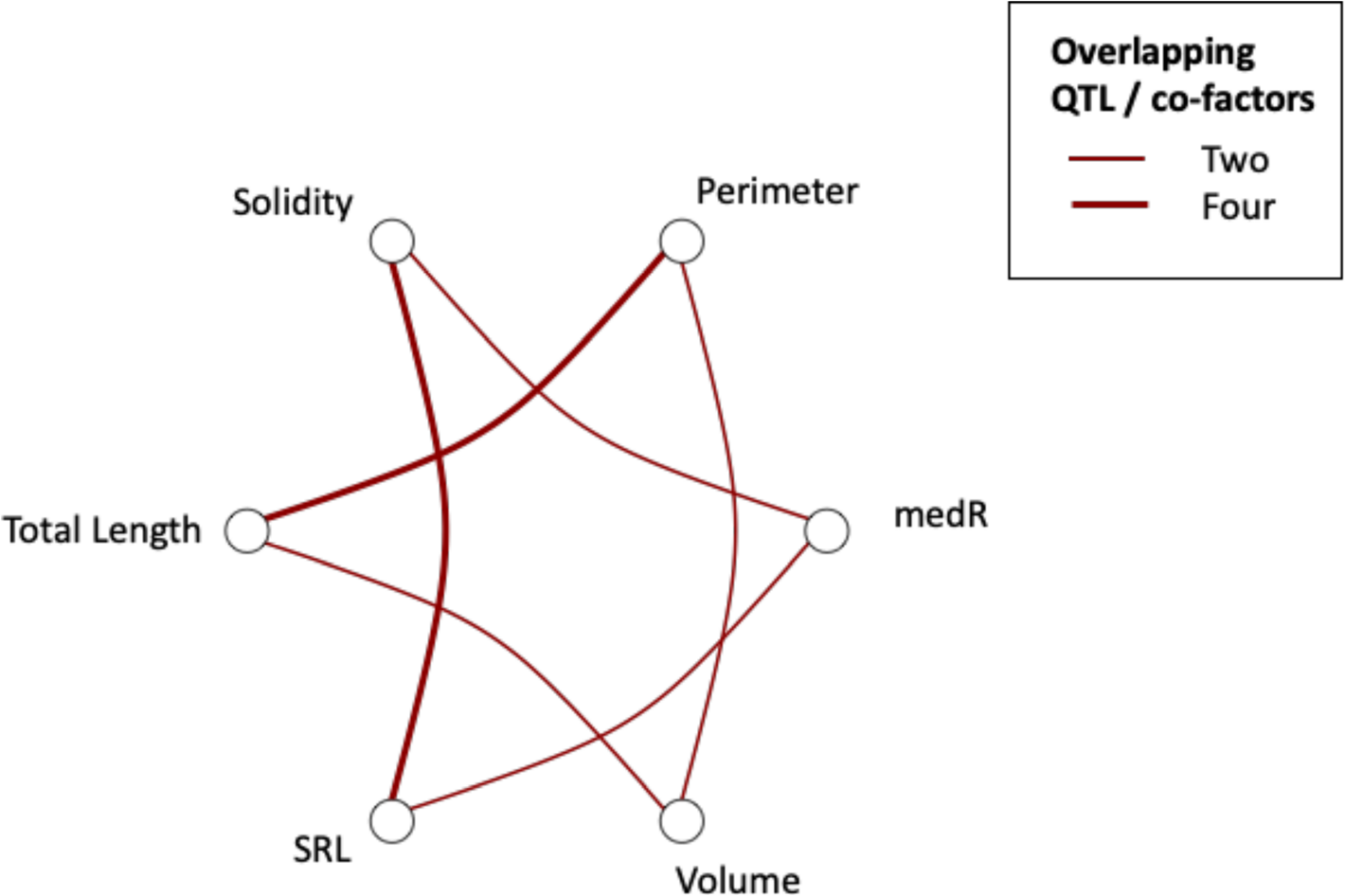
Network diagram of pleiotropic QTL association between root traits. Line thickness indicates number of shared QTL. Line width denotes number of shared QTL

**Fig. 6:**
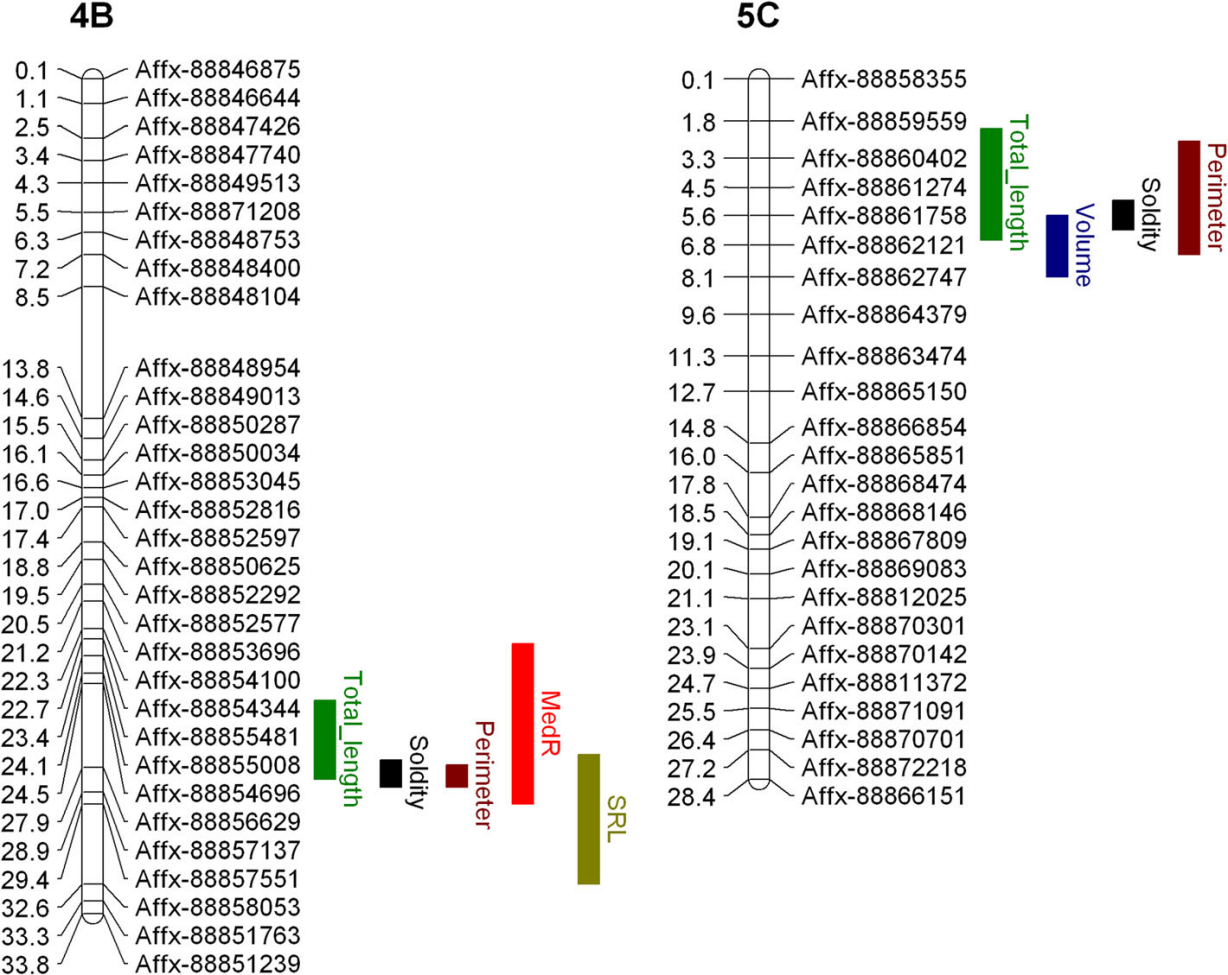
Linkage group 4B and 5C illustrating the significant overlapping QTL intervals for root architecture size metrics

A total of 16 QTL were found to be associated with 10 plant architectural traits as shown in Table 2 and Fig. 4. Of the QTL detected, 4 were observed to have an association with more than one root trait (Fig. 5). Four QTL were associated with solidity that, when combined, explain 46 % of the observed variation (Table 2). The same region on chromosome 4B was found to increase 5 root traits; specific root length (SRL), root perimeter total root length Solidity and medR (Fig. 6; Table 2). Whereas the same genetic region on chromosome 5C A focal SNP associated with medR on chromosome 2C is situated within 10 kb of the *F. vesca* gene model FvH4_2g24150.1 which encodes a protein with an auxin-binding domain (IPR000526). The focal SNP associated with medR on chromosome 5D was associated with a cluster of adjacent genes each carrying small auxin-up RNA domains typically associated with cell elongation (IPR003676). Furthermore, the *F. vesca* gene model FvH4_1g17760.1 on chromosome 1B contains Small GTPase superfamily, ARF/SAR type (IPR006689) domain. The genetic component of the speed of root growth was small, with kinetic root traits showing very low heritability scores (ranging from 0 to 5.59 %). Furthermore, no genotypic correlations between experiments were found for this trait and no associated QTL were identified.

**Table 2:** Quantitative trait locus associated with root and mycorrhizal and traits. Markers are focal single nucleotide polymorphisms representing QTL. Bold markers are identified multiple times. Only QTL with significant MQM mapping and KW & stepwise regression values are presented. Values result from MQM Mapping**.** Position in Mb scaled to the *F. vesca* genome v2.0. Co-factors are presented for each QTL where appropriate

Linkage Group	Parent	Trait	Marker Name	LOD	Position (Mb)	Effect size	*Co-factor Linkage group*	*Co-factor Marker Name*	*Co-factor Position (Mb)*	*Co-factor LOD*
6B	Redgauntlet	Arbuscles	Affx-88,816,268	3.43	4.22	10.0	*6C*	*Affx-88,888,054*	*35.73*	*3.16*
1C	Hapil	average diameter	Affx-88,869,611	4.71	3.03	11.6	*1A*	*Affx-88,810,176*	*2.06*	*3.61*
1D	Redgauntlet	average diameter	Affx-88,809,429	3.32	0.34	8.1	*1C*	*Affx-88,812,030*	*2.87*	*4.38*
2B	Redgauntlet	average diameter	Affx-88,829,957	3.58	29.22	8.8	*1C*	*Affx-88,812,030*	*2.87*	*4.38*
4A	Redgauntlet	convex area	Affx-88,849,944	4.07	4.68	10.5	*7C*	*Affx-88,901,516*	*22.44*	*2.82*
2B	Hapil	depth	Affx-88,826,056	3.09	19.97	8.4	*5C*	*Affx-88,860,094*	*2.79*	*2.83*
1B	Shared	length distribution	Affx-88,817,008	4.68	10.33	11.8	*3C*	*Affx-88,832,542*	*0.13*	*3.66*
2C	Redgauntlet	medR	Affx-88,825,906	3.44	19.73	5.01	*5C*	***Affx-88,903,845***	*6.24*	*3.19*
4B	Hapil	medR	Affx-88,856,444	5.01	27.60	12.9	*5C*	***Affx-88,903,845***	*6.24*	*3.19*
5D	Redgauntlet	medR	Affx-88,867,228	3.42	14.04	9.4	*NA*	*NA*	*NA*	*NA*
4B	Hapil	perimeter	**Affx-88,856,733**	3.78	28.04	9.4	*6A*	*Affx-88,875,604*	*8.17*	*3.34*
5C	Hapil	perimeter	**Affx-88,860,878**	5.36	5.26	19.2	*6B*	*Affx-88,874,470*	*2.74*	*3.13*
4B	Hapil	solidity	**Affx-88,857,053**	5.53	28.68	13.6	*6A*	*Affx-88,875,898*	*7.54*	*4.32*
5C	Hapil	solidity	Affx-88,903,845	4.42	6.24	12	*NA*	*NA*	*NA*	*NA*
5D	Redgauntlet	solidity	Affx-88,864,195	3.81	9.83	10.5	*NA*	*NA*	*NA*	*NA*
6C	Redgauntlet	solidity	Affx-88,887,926	4.01	35.56	9.7	*5C*	*Affx-88,903,845*	*6.24*	*4.42*
4B	Hapil	specific root length	**Affx-88,857,053**	4.87	28.68	11.9	*5C*	*Affx-88,903,845*	*6.24*	*3.72*
6A	Hapil	specific root length	Affx-88,876,002	5.33	7.32	12.8	*5C*	*Affx-88,903,845*	*6.24*	*3.72*
4B	Hapil	total length	**Affx-88,856,733**	3.09	28.04	8.6	*6A*	*Affx-88,876,441*	*6.55*	*3.46*
5C	Hapil	total length	**Affx-88,860,878**	6.42	5.26	23.8	*6A*	*Affx-88,876,441*	*6.55*	*3.46*
7B	Hapil	Vesicles	Affx-88,891,845	4.49	14.57	18.8	*7C*	*Affx-88,900,213*	*20.51*	*3.22*
5C	Hapil	Volume	**Affx-88,860,878**	6.29	5.26	22.5	*6B*	*Affx-88,874,710*	*4.81*	*5.08*

### Low phosphate tolerance QTL

A significant interaction between phosphate level and genotype was observed (*x*_(137)_^2^ = 246.2_,_*p* < 0.0001) showing that there is variation in low phosphate tolerance in the ‘Redgauntlet’ x ‘Hapil’ population. Low phosphate tolerance was determined by the relative biomass of genotypes grown under optimal and low phosphate conditions**.** There were no robust QTL associated with low phosphate tolerance observed across the two analysis.

### Arbuscular Mycorrhizal fungal association QTL

**Fig. 7:**
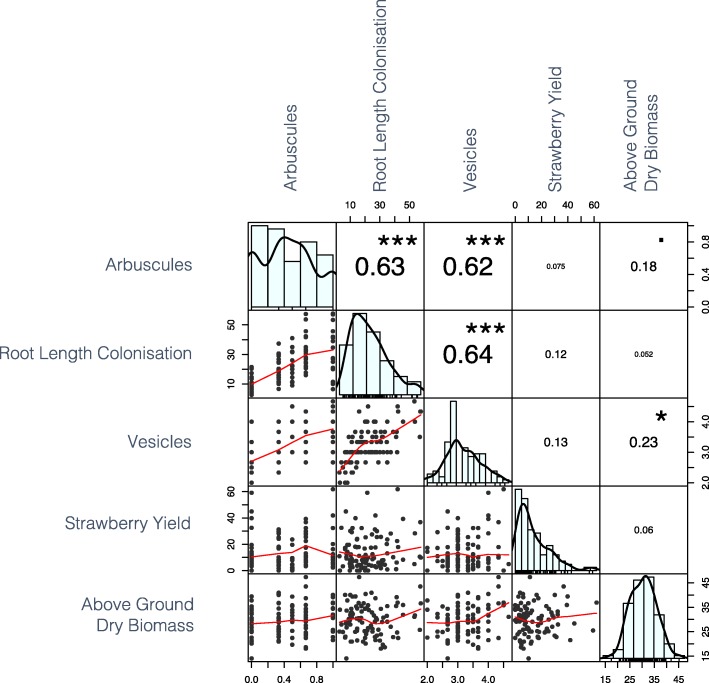
Phenotypic correlation matrix for mycorrhizal and plant traits genotype scores. Numbers represent Pearson’s correlation coefficients size of font indicates the magnitude of the value. *p values* are denoted by stars: *** < 0.001, * < 0.05, ^.^ < 0.1

Strong positive phenotypic correlations were observed between AMF traits (Fig. 7). Two robust QTL were found to be associated with AMF structures. A single locus was found to control mycorrhizal arbuscule formation on chromosome 6B whereas a locus on chromosome 7B was found to control mycorrhizal vesicle formation.

### Phenotypic and genetic correlations

**Fig. 8:**
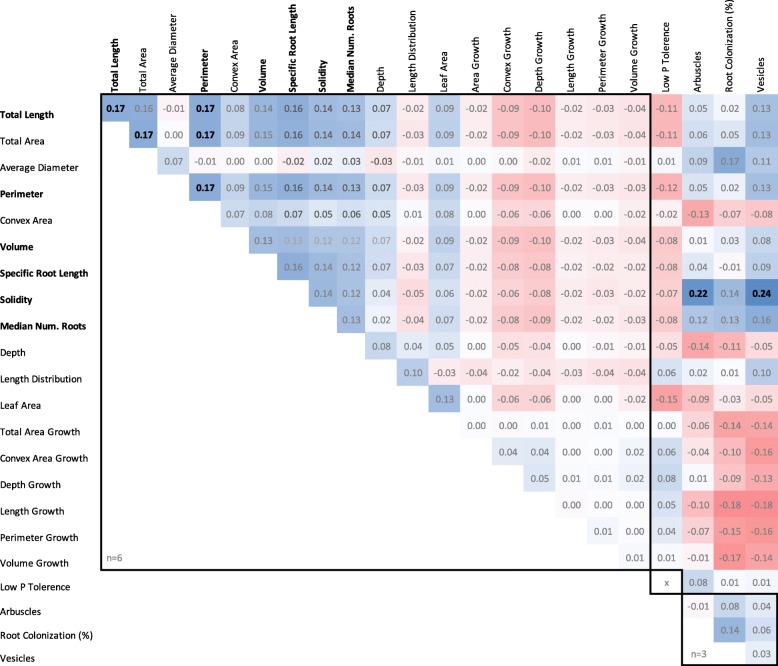
Genetic correlation between plant architecture and mycorrhizal traits. Values represent Pearson correlation coefficients. n represents number of replicates. Bold numbers are significant correlations at *p* < 0.05

Several root traits that represent metrics of root system size could be grouped as they were both phenotypically (Fig. 3) and genetically correlated (Fig. 8). This included total root length, total root area, perimeter, volume, solidity, SRL and medR, indicating these traits may be controlled by the same genetic components. This is supported by the fact that these traits (with the exception of total area) were observed to share overlapping QTL (Fig. 5). In contrast, average root diameter and length distribution were not found to be phenotypically or genotypically correlated with the root size traits. No correlation was found between low phosphate tolerance and root architecture nor between low phosphate tolerance and AMF association (Fig. 8). Interestingly, root system solidity was significantly genetically correlated with arbuscule and vesicle formation.

## Discussion

### The relationship between root system size and length distribution

We showed for the first time that a trade-off between what can be described as a “phosphate scavenging” root phenotype and root system size exists in strawberry. A “phosphate scavenging” phenotype can be defined by a high root length distribution whereby a greater proportion of horizontal roots are seen at the soil surface, as seen in bean [29]. Indeed, studies comparing root architectural trends across plant species, found seedlings of plants from nutrient rich regions had larger roots systems [33]. Similarly, a trade-off has been observed in *Senecto vulgaris* roots whereby large root systems have high exploitation but low exploitation efficiency [34]. Indeed, a maize root systems can be characterised as small and compact or large and exploratory with trade-offs between exploration and occupation resulting from phenotypic trade-offs rather than biomass restriction [35]. As no phenotypic or genotypic correlation was observed between root diameter nor root length distribution and root size traits we hypothesise that these traits are under unique genetic control.

### Root system and AMF association

Meta-analysis studies looking into the presence of a universal link between root architecture and mycorrhizal association have found mixed results. A negative relationship has been observed between mycorrhizal dependency (the positive impact of AMF) and total root length across different plant species [36]. Furthermore, research looking into the degree to which AMF alter host root systems have found greater root modification potential in plants with the highest mycorrhizal dependency [37]. By contrast, another study across multiple species found a coarser root architecture does not lead to a greater mycorrhizal colonisation nor growth benefit from association [38]. Here, a higher solidity was genetically correlated with a greater frequency of arbuscule and vesicle AMF structures. A high solidity can be described by “dense roots growing together which thoroughly explore the root network area” [39]. Similar studies in rice have found a negative relationship between thoroughness of the root system (solidity) and the extent of the root system (convex area) [39], whereby a dense root system can be said to explore a smaller area of soil and thus stand to benefit more from exploiting an AMF extraradicular network. However, such a conclusion cannot be drawn here as we see no relationship between solidity and convex area across our genotypes.

### Benefits of AMF colonisation

Greater dry matter and phosphate uptake have been observed in strawberry plants colonised with mycorrhiza under low phosphate conditions [16]. Further than this, application of AMF to the strawberry cultivar ‘Elsanta’ was found to result in a greatly improved yield in coir production [13]. Here, we saw a positive correlation between above-ground dry biomass and AMF vesicle formation amongst genotypes (Fig. 7). It may be speculated that a plant with a larger photosynthetic area has greater carbon availability for symbionts, and that this may lead to increased numbers of AMF carbon storage structures - vesicles. Such a relationship may indicate a benefit to the AMF as opposed to a benefit for the plant. Unfortunately, no positive relationship was observed between strawberry yield and AMF association across ‘Redgauntlet’ x ‘Hapil’ genotypes grown in Terra-Green® (Fig. 7) indicating that AMF strawberry interactions are complex and that any associated benefits may be both substrate and genotype specific. The use a calcined attapulgite clay growth media Terra-Green® results in the adsorption of labile phosphate and enhances the necessity of mycorrhizal associations to access soil phosphate [40]. As such the observed AMF associations in the current experiment may be higher than that of other commercially relevant growth media.

### Parental root architecture and propagation method

We found greater resolution to discriminate differential root architecture in the two parental cultivars when root systems were developed through ‘pinning down’ plantlets as opposed to misted tip propagation. Pinned down plantlets were able to establish a root system whilst attached to the mother plant and thus had access to parental nutrients. It is known that root architecture can be altered in response to soil nutrient status [28, 41]. Therefore, the misted tip propagation method may have led to an altered root system architecture through the absence of parental nutrients. However, low internal phosphate status was not found to trigger the phosphate starved morphological changes observed in low phosphate external environments [42]. Research investigating the performance and nutrient use efficiency of plants developed using different propagation strategies may have downstream implications for best practice nursery propagation. In contrast to the findings above, parental average root diameter was found to vary consistently in both experiments and we also observe that average root diameter appears to be controlled independently from the root traits representing size (Fig. 8).

### Hormonal control of root architecture

The ethylene and auxin pathway have been identified as key regulators of root branching, root hair elongation and low phosphate detection [43–45]. Two of the identified QTL associated with medR, co-localised with core genes in the auxin pathway of *F. vesca* diploid strawberry. Interestingly the length distribution QTL on chromosome 1B was associated with a gene containing a small GTPase superfamily, ARF/SAR type domain, functional studies in *Arabipdosis* have shown this domain to play a role in root hair tip initiation [46].

### Heritability of traits and genetic correlations

Heritability scores for all investigated traits were low (5–14.5%; Supp. Table 2), indicating a large environmental component controlling variation in the assessed traits. Arbuscules have a heritability of 0%. The low arbuscule heritability is an artefact of low arbuscule occurrence across 100 transects, with 0, 1 or 2 arbuscules found in each root system. The mean values for arbuscules provided a more quantitative score for QTL assessment. In spite of the low heritability scores, 18 QTL controlling traits were identified with each explaining between 5.9 and 13.4% of the observed phenotypic variation. Low genetic correlation between root traits can be explained by the highly plastic nature of roots which can alter in response to multiple abiotic factors including nutrient concentration, water availability, oxygen content, soil density and pH [3].

### QTL co-localisation

We observed a network of pleiotropic QTL controlling multiple root size traits in strawberry indicating several regions of the genome are important for root architecture. Similar studies have identified regions of the genome associated with multiple root architecture in both rice and maize [35, 39].

### Phosphate tolerance and root architecture

No association between low phosphate tolerance and length distribution was observed in this study. Indeed, no genotypic correlation was observed between the root architectural traits observed under optimal phosphate conditions and low P-tolerance. However, roots have been shown to exhibit phenotypic plasticity in response to low phosphate environments, whereby phosphate starved plants show reduced primary root growth, reduced growth rate due to lower phosphate metabolism and increased lateral branching [30, 47]. In this experiment root architecture was measured under optimal growth conditions. Thus, it is likely that any improvement in low phosphate tolerance is neither mediated nor represented by the root architecture of a genotype when grown under optimal phosphate conditions. Further work should determine whether ‘Redgauntlet’ and ‘Hapil’ root architecture is altered under low phosphate conditions and whether specific low-P root traits are associated with enhanced P-tolerance. Furthermore, we can determine whether root plasticity itself is associated with an increase in low P-tolerance in strawberries.

## Conclusions

Understanding the genetic control of root architecture can guide breeding strategies towards developing optimal root systems. Here we describe a high throughput root phenotyping platform for strawberry which allows quantification of the “hidden” below-ground component of plant variation. We show that different components of root architecture: root system size, length distribution and root diameter are under different mechanism of genetic control. Low P-tolerance was not found to be phenotypically or genotypically associated with root architecture traits in strawberry plants when grown under optimal nutrient conditions. Future work must characterise whether root plasticity under low phosphate conditions is associated with heightened phosphate acquisition in strawberry.

## Methods

### Root architecture quantification

#### Plant material

The strawberry (*Fragaria x ananassa*) ‘Redgauntlet’ x ‘Hapil’ mapping population was used to study root architecture, AMF association and low phosphate tolerance. Plant material was generated at NIAB EMR, the F1 cross was initially made to study *Verticillium dahliae* resistance [48]. For the initial assessment of parental root architecture, 10 runner plants of ‘Redgauntlet’ and ‘Hapil’ were pinned down into rhizotrons. All plantlets remained attached to the mother plants during root system development. Due to the large scale of the experiment required for assessment of population root system architecture, 168 genotypes and parental plants were propagated as misted tips. The misted tips were cut from genotypes and inserted into rhizotrons containing 1 L peat soil sieved to 5 mm. One plant per genotype was grown in each rhizotron. After 1 week at 80% humidity, the rhizotrons were randomised and transferred into glasshouse conditions of 16:8 h; 22:16 °C day: night, 60 RH%. Two 4 L/h drippers per rhizotron supplied irrigation at 16 ml / d (+/− 2%) for 30 s 4 times per day with added strawberry nutrient feed; Solufeed SF-C (N:P:K, 8:12:35 + 4MgO). The experimental layout was a randomised block design with blocks from South to North. The experiment was repeated three times with two replicates per time point.

#### Rhizotron construction

Rhizotron containers were made from two clear acrylic sheets (24 × 20 × 0.4 cm; Plexiglas®), acrylic spacers (0.5 cm; Acrylic Online, Hull, UK) and were held together by 5 cm fold-back clips. Opaque vinyl covers prevented light from entering rhizotrons. Modified crates (50 × 30 × 25 cm) supported the rhizotrons at an angle of 25° to promote root growth along the rhizotron front sheet.

#### Root imaging

An imaging rig was constructed (80 × 70 × 133 cm) to allow simultaneous root and shoot imaging. Cameras were fixed 1 m from the rhizotron surface and 65 cm above the plant canopy. Images were taken with an 18-megapixel full-frame digital single-lens reflex camera (Canon; EOS 1200D) equipped with an 18–55 mm lens (Canon EFS). Illumination was provided by LED-panels with constant illumination. Two cameras were controlled remotely by one laptop with EOS Utility software (Canon, USA Inc., Lake Success, NY) to trigger simultaneous image capture. The minimum detectable size of the colour 24-bit RGB image was ~ 0.1 mm pixel^− 1^. The resolution of images (230 μm per pixel) could distinguish fine scale strawberry roots. Root and shoot images were taken simultaneously over 6 time points between 7 and 21 days after plant establishment.

#### Image analysis

Image analysis software was developed in C++ for QR decoding, image pre-processing and quantification of root architecture traits, which can be obtained from https://github.com/eastmallingresearch/Image-processing/tree/master/C%2B%2B/root_architecture.

#### Image pre-processing

Below-ground images were converted to greyscale. Adaptive thresholding used the mean neighbourhood area of each image as a threshold value to correct for uneven illumination and rhizotron surface reflection. Noise on resulting binary images was removed with an arbitrary threshold of the contour size. Pre-processing removed the majority of background pixels; however manual noise removal was required as some root pixels were disconnected from the main root structure and thus smaller than the deselection threshold. Above ground images were converted into HSV colour space and global thresholding was applied on the hue channel to extract the canopy from background. Canopy area was calculated by quantifying the pixel number corresponding to plant leaves.

#### Quantification of root architecture

Root architecture traits were calculated in pixel values including total root length, average diameter, root area, root perimeter, convex area, solidity (network area divided by the convex area), depth, median number of roots (MedR), specific root length (SRL) and length distribution. Total area was the root pixel number calculated using the binary image. Total root length was calculated as in Kimura et al. [49], such that the number of orthogonal and diagonal connected pairs in the skeleton image were accounted for in the calculation to minimize confounding effects of sample orientation and root overlap. This method was extended to quantify the root length distribution by calculating the ratio between the root length in the upper third and lower two-thirds of the root system [50]⁠. The distance transformation was applied to the binary rhizotron image, and the grey level intensities of pixels indicating the minimum distance to the nearest boundary. After distance transformation, root radius could be obtained by extracting the intensity of each pixel corresponding to the root skeleton and thus used to calculate the volume, average diameter and SRL [50]⁠. Root perimeter, solidity, depth, convex area, depth and MedR were calculated based on the binary image as lyer-Pascuzzi [50]⁠. Root growth rate was calculated as the decay rate of the exponential fit over time points 2 to 5. The growth rate was measured using total root length, total root area, convex area, perimeter and volume.

### Low phosphate tolerance

Low phosphate tolerance was measured in the ‘Redgauntlet’ x ‘Hapil’ mapping population. Pinned down, cold stored (− 2 °C) strawberry plants were transplanted into 2 L square pots containing coir (Botanicoir, England). Plants were arranged in a complete randomized block design with 4 replicate plants per 173 genotypes across the two fertigation treatments. Automated fertigation was supplied through drippers providing optimal phosphate (N:P:K 176:36:255 ppm) or low phosphate (N:P:K 176:10:255 ppm) fertigation at 1 kg l^− 1^ (rate: 10 s every 45 minutes). Preliminary phosphate dose experiments were used to determine deficit fertigation rates based on reduction in plant biomass production. Fertigation was supplied at a rate of 1 min six times per day through 4 L/h drippers. At 146 days after removal from cold storage, above ground and root plant material was harvested, oven dried for 7 days at 80 °C and dry biomass was quantified. Low phosphate tolerance was calculated by the relative difference between plants of each genotype grown under optimal and low phosphate conditions. Mixed models were compared to test for an interaction between genotype and phosphate levels using a Chi-square likelihood ratio test.

### AMF association

The propensity for genotypes to form mycorrhizal association was quantified in the ‘Redgauntlet’ x ‘Hapil’ mapping population. The randomized block experimental design contained three replicate plants per 147 genotypes. Glasshouse conditions were 16:8 h day: night, 20:14 °C. Pinned down, cold stored (− 2 °C) plants were transplanted into 2 L pots containing Terra-Green®. Before transplanting roots were trimmed by 1–2 cm. The following AMF inoculum was added to the planting hole for each plant: 15 g granular commercial mix of five mycorrhizal species (*Claroideoglomus claroideum, Glomus microagregatum, Rhizophagus irregularis, Funneliformis mosseae and F. geosporus, “Rootgrow”* propagation mix 2; PlantWorks Ltd., Kent, UK). Plants were irrigated by hand for 1 month after which plants were fertigated using Vitex Vitafeed (N:P:K, 1:0:2, 18:0:36) at 1 kg l^− 1^ (rate: 10 s every 45 minutes). Fruit size and marketable yield were assessed twice a week from 52 d after removal from cold storage. After 95 days, plants were destructively harvested, above ground dry biomass was quantified and root samples were taken for analysis. Roots were cleared in 10% KOH and stained with Trypan Blue. Root length colonisation (RLC%) was quantified using a dissection microscope where hyphal, arbuscule and vesicle presence were scored across 100 horizontal and vertical intersects of a 1 cm grid [13, 51].

#### Linkage map generation

DNA was extracted from leaf material using the plant Qiagen DNAeasy plant mini extraction kit. DNA samples were genotyped using the Istraw90 Affymetrixs chip containing 138 k probe sets. SNP data can be found in Supp. File 1. The ‘Redgauntlet’ x ‘Hapil’ linkage map was created using the Crosslink program [52] designed for Octoploid linkage map development. Segregating markers from five bi-parental strawberry populations were combined to make the consensus map as detailed in [52].

#### Quantitative trait loci (QTL) analysis

Genetic analysis was undertaken using R Version 3.5.1 [53]. QTL mapping of phenotypic traits was performed through Kruskal–Wallis analysis on mean genotype trait values to identify focal single nucleotide polymorphisms (SNPs). The most significant marker was selected for each QTL and then combined into selection through a stepwise linear regression model using a stepwise regression function [54]. Interval mapping and MQM mapping was conducted in MapQTL® [55]. Potential co-factors were identified through a two-step process: first significant QTL were treated as co-factors to identify putative interacting loci, then the reciprocal analysis was preformed treating these newly identified loci as co-factors, cofactors were retained if they improved the LOD score of initial QTL. QTL identified in both analysis are considered to be robust and thus reported here. Heritability and proportional reduction of error was calculated as specified in Cockerton et al., 2019 [48]. Principal component analysis was used to determine the components accounting for the largest proportion of variation in genotypes. Genetic correlations were calculated through in-house scripts, through taking the average Pearson’s correlation coefficient between each reciprocal replicate. Between experiment genetic correlations were calculated through Pearson’s correlation. Phenotypic correlations were quantified using R package [56]. The Network diagram was created to depict the number of overlapping QTL and co-factors using R package “network” [57] and “visNetwork” [58]. The intersect function of Bedtools was used to identify QTL locations within 10 kb of *Fragaria vesca* genome v.4 gene models [59]. Functional annotations of *F. vesca* gene models detailed in were generated using Interproscan v.4.

## Supplementary information

**Additional file 1: Supplementary Table 1.** Trait means of the root architectural traits of two parental cultivars and F1 genotypes. CV is the coefficient of variation. Significance values associated with ANOVA tests. Units are pixel number unless otherwise stated. SRL – Specific root length, medR- medium root number. *p values* are denoted by stars: *** < 0.001, ** < 0.01, * < 0.05. Values are provided in pixel number or relative statistics. The difference among F1 genotypes is calculated without parents.

**Additional file 2: Supplementary Table 2.***H*^2^ is broad-sense heritability associated with each phenotyping event. SRL – Specific root length, medR- medium root number.

**Additional file 3.**

## Data Availability

The SNP datasets analysed as part of this study can be found in supplementary file 1. The remaining datasets during the current study are available from the corresponding author on reasonable request.

## Abbreviations

AMF: Arbuscular Mycorrhizal Fungi
i35k: Istraw35 Affymetrix chip
i90k: Istraw90 Affymetrix chip
MedR: Median number of roots
PCA: Principal Component Analysis
PUE: Phosphorus Use Efficiency
QTL: Quantitative Trait Loci
QR: Quick Response
RH%: Percentage Relative Humidity
RLC%: Root Length Colonisation
RGB: Red Green Blue
RSA: Root System Architecture
RxHa: ‘Redgauntlet’ x ‘Hapil’ mapping population
SNP: Single Nucleotide Polymorphism
SRL: Specific Root Length
