# Supplementary material for: Genetic and phenotypic associations between root architecture, arbuscular mycorrhizal fungi colonisation and low phosphate tolerance in strawberry (Fragaria × ananassa)
Source: BMC Plant Biol. 2020 Apr 9;20:154. doi: 10.1186/s12870-020-02347-x (PMC7146916; doi:10.1186/s12870-020-02347-x)
Supplement: Supplementary file 1 — Additional file 1: Supplementary Table 1. Trait means of the root architectural traits of two parental cultivars and F1 genotypes. CV is the coefficient of variation. Significance values associated with ANOVA tests. Units are pixel number unless otherwise stated. SRL – Specific root length, medR- medium root number. p values are denoted by stars: *** < 0.001, ** < 0.01, * < 0.05. Values are provided in pixel number or relative statistics. The difference among F1 genotypes is calculated without parents. [file 12870_2020_2347_MOESM1_ESM.docx]

**Supplementary Table 1:** Trait means of the root architectural traits of two parental cultivars and F1 genotypes. CV is the coefficient of variation. Significance values associated with ANOVA tests. Units are pixel number unless otherwise stated. SRL – Specific root length, medR- medium root number.  *p values* are denoted by stars: *** < 0.001, ** < 0.01, * < 0.05. Values are provided in pixel number or relative statistics. The difference among F1 genotypes is calculated without parents.

| **Trait** | **Hapil** | **Redgauntlet** | **F1** | **CV(%)** | **Difference among parents** | **Difference among F1 genotypes** |
| --- | --- | --- | --- | --- | --- | --- |
| Total length | 15869 | 13768 | 17536 | 54.9 | ns | *** |
| Total area | 12318 | 9413 | 12777 | 54.5 | ns | *** |
| Average diameter | 0.922 | 0.841 | 0.894 | 8.79 | * | * |
| Perimeter | 7815 | 6498 | 8437 | 54.7 | ns | *** |
| Convex area | 315880 | 284390 | 34934 | 37.3 | ns | ** |
| Volume | 93580 | 83032 | 95240 | 31.4 | ns | *** |
| SRL (Length: Area) | 0.166 | 0.161 | 0.1727 | 29.3 | ns | *** |
| Solidity  (Convex Area: Area) | 0.0391 | 0.0333 | 0.0370 | 38.2 | ns | *** |
| medR (Frequency) | 27.3 | 22.5 | 27.1 | 31.3 | ns | *** |
| Depth | 780.3 | 807.3 | 788.1 | 19.4 | ns | *** |
| Length distribution  (Root area top 1/3: Root area bottom 2/3) | 0.303 | 0.320 | 0.305 | 35.9 | ns | *** |
| Leaf area | 17438 | 27827 | 30896 | 56.8 | * | *** |
| Perimeter growth (%) | 7.134 | 2.86 | 4.37 | 124 | ns | * |
| Area growth (%) | 4.43 | 2.33 | 3.69 | 137 | ns | * |
| Length growth (%) | 8.03 | 2.95 | 4.50 | 130 | ns | * |
| Arbuscule | 0 | 0.67 | 0.40 | 73.6 | ns | ns |
| Vesicles | 1.67 | 3.67 | 3.01 | 22.6 | ns | ns |
| RLC% | 6 | 16 | 21.6 | 58.6 | ns | ** |
| Total Yield (g) | 50.2 | 17.9 | 14.5 | 87.8 | ns | *** |
| Dry Biomass (g) | 30.3 | 31.9 | 28.9 | 20.5 | ns | *** |
| Phosphate Tolerance | 0.04 | 0.79 | 0.15 | 2.5 | NA | *** |
