# Supplementary material for: Genetic and phenotypic associations between root architecture, arbuscular mycorrhizal fungi colonisation and low phosphate tolerance in strawberry (Fragaria × ananassa)
Source: BMC Plant Biol. 2020 Apr 9;20:154. doi: 10.1186/s12870-020-02347-x (PMC7146916; doi:10.1186/s12870-020-02347-x)
Supplement: Supplementary file 2 — Additional file 2: Supplementary Table 2.H2 is broad-sense heritability associated with each phenotyping event. SRL – Specific root length, medR- medium root number. [file 12870_2020_2347_MOESM2_ESM.docx]

**Supplementary Table 2:** *H*^2^ is broad-sense heritability associated with each phenotyping event. SRL – Specific root length, medR- medium root number.

| **Trait** | **H^2^** |
| --- | --- |
| Total length | 14.0 |
| Total area | 14.4 |
| Average diameter | 5.0 |
| Perimeter | 13.6 |
| Convex area | 6.3 |
| Volume | 12.0 |
| SRL | 10.1 |
| Solidity | 11.8 |
| medR | 11.3 |
| depth | 7.2 |
| Length distribution | 7.7 |
| Leaf area | 11.0 |
| Arbuscules | 0.0 |
| Vesicles | 14.5 |
| Root length colonisation | 14.0 |
| Low phosphate tolerance | NA |
